# Determinants of adolescent pregnancy in indigenous communities from the Peruvian central jungle: a case–control study

**DOI:** 10.1186/s12978-021-01247-z

**Published:** 2021-10-12

**Authors:** Jhonatan R. Mejia, Ángel J. Quincho-Estares, Asstrid J. Flores-Rondon, Giancarlo Reyes-Beltran, Irene L. Arias-Sulca, Estephanie Palomino-Hilario, Jessica E. Barrientos-Cochachi, Carlos J. Toro-Huamanchumo

**Affiliations:** 1 Universidad Nacional del Centro del Perú, Sociedad Científica de Estudiantes de Medicina del Centro, Huancayo, Peru; 2 Universidad San Ignacio de Loyola, Unidad de Investigación Para la Generación y Síntesis de Evidencias en Salud, Lima, Peru

**Keywords:** Pregnancy in adolescence, Indigenous population, Social determinants of health, Case–control studies, Peru

## Abstract

**Background:**

Adolescent pregnancy carries a high risk of severe health issues for both the mother and the newborn. Worldwide, 21 million adolescents give birth every year, with high percentages in Latin America. Most of the risk factors are met in indigenous communities, which is an underrepresented and poorly studied population. We aimed to assess the determinants of adolescent pregnancy in indigenous communities from the Peruvian central jungle.

**Methods:**

Through a case–control study, female adolescents aged 13 to 19 years old from seven indigenous communities of the Peruvian central jungle were interviewed. Adolescents with (cases) and with no (controls) pregnancy history, such as current pregnancy, children and abortion, fulfilled our eligible criteria. Our instrument explored: sociodemographic, adolescent and family characteristics, as well as perceptions of adolescent pregnancy. We performed a penalized maximum likelihood logistic regression analysis to obtain Odds Ratios (OR) and their 95% confidence intervals (95% CI).

**Results:**

We enrolled 34 cases and 107 controls. Overall, 53.9% were 15 to 19 years old. We found a significant association of being 15–19 years old (OR = 6.88, 95% CI 2.38–19.86, p < 0.0001) and an elementary school level of instruction (OR = 5.59, 95% CI 1.95–16.06, p = 0.001) with the risk of adolescent pregnancy. A marginal statistical significance between having five to six siblings and adolescent pregnancy was also reported (OR = 2.70, 95% CI 0.85–8.61, p = 0.094). Furthermore, adolescents with sexual and reproductive health communication with parents had a lower risk of adolescent pregnancy (OR = 0.17, 95% CI 0.06–0.47, p = 0.001).

**Conclusion:**

Our results suggest that public health and educational efforts should be age-specific focused within indigenous communities of the Peruvian central jungle, encouraging parents to talk about sexual and reproductive health topics with adolescents.

**Supplementary Information:**

The online version contains supplementary material available at 10.1186/s12978-021-01247-z.

## Plain language summary

Worldwide, 21 million adolescents give birth every year, with high percentages in Latin America and rural areas. Indigenous communities are mainly located in rural areas and are exposed to multiple risk factors of adolescent pregnancy. We aimed to find the factors that have an influence on adolescent pregnancy in indigenous communities from the Peruvian central jungle. We conducted a case–control study identifying female indigenous adolescents from the Peruvian central jungle with or without pregnancy history. Our survey explored sociodemographic, adolescent and family characteristics, as well as perceptions of adolescent pregnancy. Being 15 to 19 years old, having an elementary educational level, and five to six siblings increased the risk of adolescent pregnancy. On the other hand, adolescents with sexual and reproductive health communication with parents had a lower risk of adolescent pregnancy. Furthermore, eight out of ten adolescents opposed to sexual intercourse at an early age. Public health and educational efforts should be age-specific focused within indigenous communities of the Peruvian central jungle, encouraging parents to talk about sexual and reproductive health with adolescents.

**Supplementary Information:**

The online version contains supplementary material available at 10.1186/s12978-021-01247-z.

## Introduction

About 21 million young women under 19 years old give birth every year worldwide [1]. The highest percentages come from developing regions, for instance, Africa, where adolescent pregnancy ranges from 60 to 80% [2–4]. Percentages are lower in Latin America, but they still represent a large proportion of female adolescents, with a prevalence that varies between 27 and 41% [5].

Adolescent pregnancy is associated with several factors that, when avoided, may prevent severe health issues. In rural areas, the low socioeconomic status and the lack of access to education are commonly associated with adolescent pregnancy [3, 6]. Along with sociodemographic characteristics, family history, such as a close relative’s previous experience of adolescent pregnancy [7], domestic violence [8], and even sexual harassment [9] are also factors associated with adolescent pregnancy.

The social environment has an important influence on the early onset of sexual intercourse, highlighting the social pressure [9], drugs use, and alcohol consumption [10, 11] that contribute with an increased risk of accumulating multiple sexual partners among adolescents [9]. As a result of this exposure, adolescent mothers may have reiterated pregnancies during their adolescence [12], with pregnancy intervals of 24 to 45 months [13]. This might contribute to severe consequences related to newborn and maternal health, such as preterm birth [14], low birth weight [15], hypertensive disorders [16], maternal [17] and perinatal mortality [16].

The indigenous population all over the world reaches 370 to 500 million, representing vast cultural diversity. Nevertheless, they are one of the most discriminated groups [18]. In Peru, they are placed in the Amazon, counting for approximately one-third of a million. From this group, about 50% are under 15 years old [19]. Most of the adolescent pregnancy’s risk factors are met in indigenous communities, where people are exposed due to sociodemographic barriers. We considered it necessary to provide an approximation to this reality because they are an underrepresented, neglected, and poorly studied population. Therefore, we aimed to assess the determinants of adolescent pregnancy in indigenous communities of the Peruvian central jungle.

## Materials and methods

### Study design and setting

**Fig. 1:**
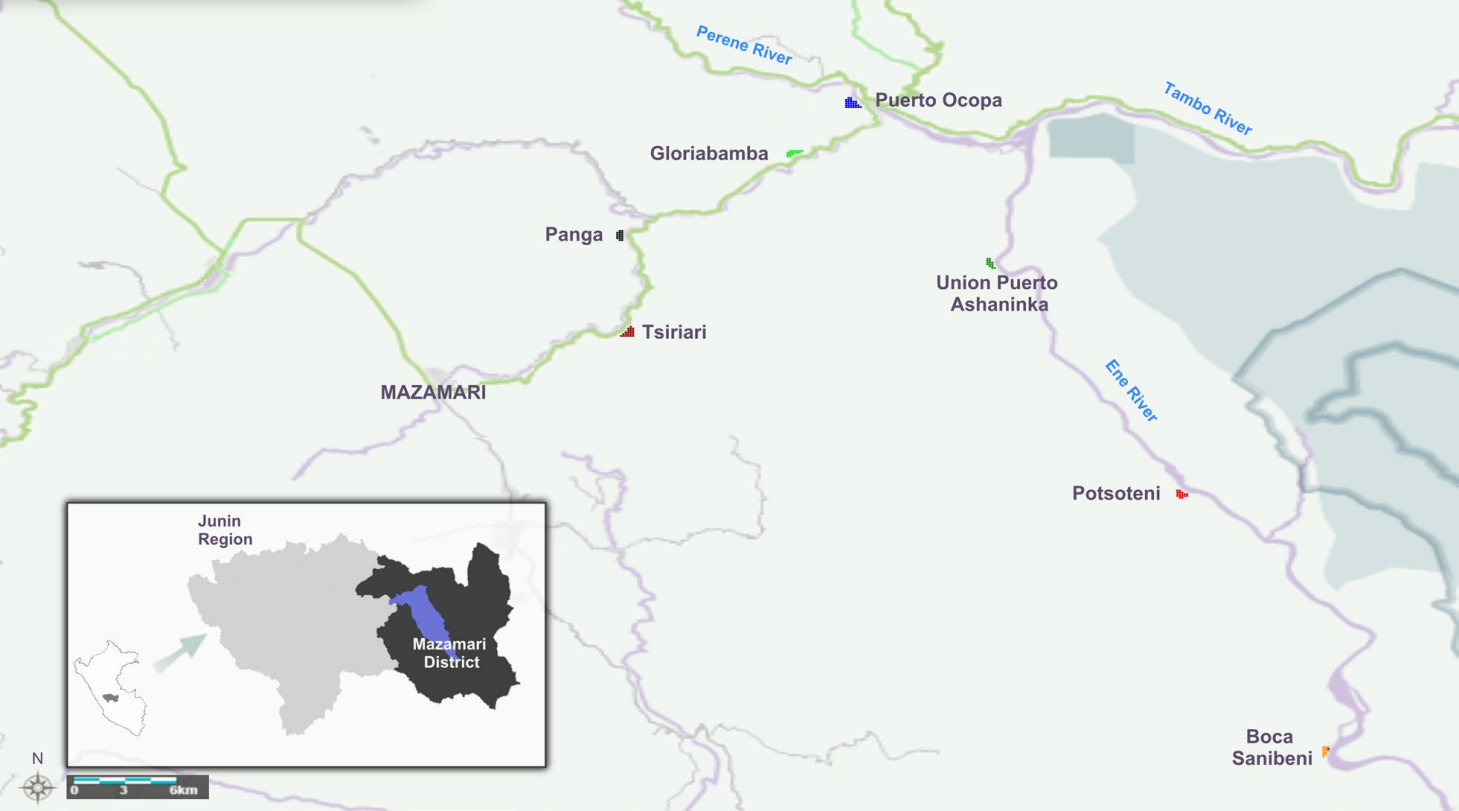
Map of the study area. This figure illustrates Junin region (gray colored), Satipo province (dark colored) and its capital city, Mazamari (blue colored). Indigenous communities are signalized with different colors next to highways and rivers

We conducted a case–control study on August–November 2018 and July 2019, in the Peruvian central jungle. We visited seven Ashaninka communities of Satipo, province of Junin, Peru (Fig. 1). These communities are located 20 min to 5 h away from Mazamari, the main city of Satipo. Tsiriari (20 min, 14 km), Panga (30 min, 24 km), Gloriabamba (45 min, 38 km), and Puerto Ocopa (an hour and a half, 45 km) are commonly accessible by car. Access to Unión Puerto Ashaninka (2 h, 66 km), Potsoteni (3 h, 89 km), and Boca Sanibeni (5 h, 118 km) is by boat. Communities’ dwellers consider themselves as Ashaninkas because of their parents’ origins, native language and traditions. Most of them are cacao, coffee, and fruit farmers. Unfortunately, the vast majority live in extreme poverty lacking essential services, such as electricity, drinking water, drainage system, health care and clothing. Malnutrition is the most frequent non-communicable disease, and dengue is the most common vector-borne viral disease [20].

### Population and sample

Female Ashaninka adolescents from the localities previously described were our population. Adolescents aged 13 to 19 years old with (cases) and with no (controls) pregnancy history, such as current pregnancy, children, and abortion, fulfilled our eligibility criteria. We were unable to ascertain a case through obstetric registries due to the lack of them at community level. Their denial to participate in the study was respected and considered as an exclusion criterion. Assuming a proportion of exposure in cases and controls of 78.8% and 54.3% controls, respectively [21], a power of 80% and 5% of significance, we required 37 cases and 111 controls (3 controls per 1 case). We used a convenience sample due to the lack of obstetric registries in all the localities, a fact that did not allow us the possibility of randomization.

### Process

Three trips were necessary to reach our sample. The first one took place during the XII National Multidisciplinary University Research and Service Camp (CUMIS)—Mazamari 2018, organized by the Scientific Society of Medical Students from the Center of Peru (SOCIEMC). On this trip, we visited Boca Sanibeni, Potsoteni and Union Puerto Ashaninka. Three authors were in charge of data collection being helped by three CUMIS participants previously trained. Adolescents were recruited during primary health care activities and through house-to-house interviews. The second and third trips were performed by six authors and a collaborator in Tsiriari, Panga, Gloriabamba, and Puerto Ocopa. Cases and controls were recruited at schools and house to house.

We developed and pilot-tested a survey based on a previous study [21], which was applied through an interview. The instrument comprised the following sections: (1) general data, (2) adolescent’s characteristics, (3) family characteristics, (4) adolescent mother’s characteristics, and (5) social characteristics. Participants were interviewed in comfortable and private environments. If the adolescent was not fluent in Spanish, a member of the community with fluency in Spanish helped us and serve as a translator, a fact that was little used (five interviews). We were at risk of social desirability bias because of the private questions and the sociocultural context that limit adolescents to talk about sexual life outright. To overcome this potential bias, our questionnaire had redundant questions, interviewers tended to give a sense of confidence, and surveys were saved in an amphora after each interview to protect confidentiality.

### Variables

Our primary outcome was adolescent pregnancy. We looked for the associated factors exploring individual, family, adolescent’s mother, and social characteristics. Age was dichotomized according to adolescence stages (10 to 14 years old as early adolescence, and 15 to 19 years old as late adolescence) [22]. We asked for the current educational level of the adolescent and the highest level reached by parents (none/elementary/high-school/technician-university). The socioeconomic status was categorized (A–B = 690–1170 USD/C = 300–690 USD/D–E < 300 USD) according to the 2017 National Household Survey performed by the Peruvian National Institute of Statistics and Informatics [23].Parents’ history of being an adolescent mother or father was considered as a Yes/no question, as well as the family history (adolescent’s mother or sister) of adolescent pregnancy. We asked for the position within siblings, establishing three categories (youngest/middle/oldest). Similarly, we collected the number of siblings, which was categorized into tertiles (1–4/5–6/7–12). We also asked for the place of birth (native/foreign), occupation (none/study/work/study and work), economic dependence (herself/family/couple/family and couple), cohabiting (living alone or with family/couple/family and couple), age of menarche, age at first sexual intercourse, adolescence stage at first sexual intercourse (early/late adolescence), number of sexual partners, use of contraceptive methods, frequency of contraceptive use (never/sometimes/always), type of contraceptive method (none/barrier/hormonal/barrier and hormonal), desired pregnancy, previous abortion, number of pregnancies, sexual and reproductive health communication with parents (defined as parent-adolescent talks about risk sexual behavior or contraceptive methods) [24], and adolescent couple’s sociodemographic characteristics.

### Analysis

We constructed a database in Microsoft Excel. For quality control and completeness of data we used a double-data entry and independent resolution of any discrepancies. No questionnaires with missing information were included. Final analyses were performed in Stata v14 (StataCorp LP, College Station, TX, USA).

We used frequencies and percentages to describe categorical variables, whereas means with standard deviation (SD), and median with interquartile range (IQR) for normal and non-normal numerical variables, respectively. We used the chi-squared and Fisher exact tests for categorical variables in the bivariate analysis.

Logistic regression analyses were performed to identify the determinants of adolescent pregnancy. According to epidemiological plausibility and to address estimation bias from adolescent pregnancy having a low prevalence in our sample, we restricted the number of independent variables in multivariable analysis to eleven. As proposed by Firth, we estimated our regression models using penalized likelihood to reduce small-sample bias in maximum likelihood estimation [25] and to manage the presence of sparse data [26]. To identify the determinants of adolescent pregnancy, we performed a manual forward selection method using likelihood ratio tests until reaching a parsimonious multivariable model. We presented our results as odds ratios (ORs) with their 95% confidence intervals (95% CI). A p-value below 0.05 was considered statistically significant. Finally, we performed a post hoc power analysis for the variables included in the parsimonious model.

## Results

**Fig. 2:**
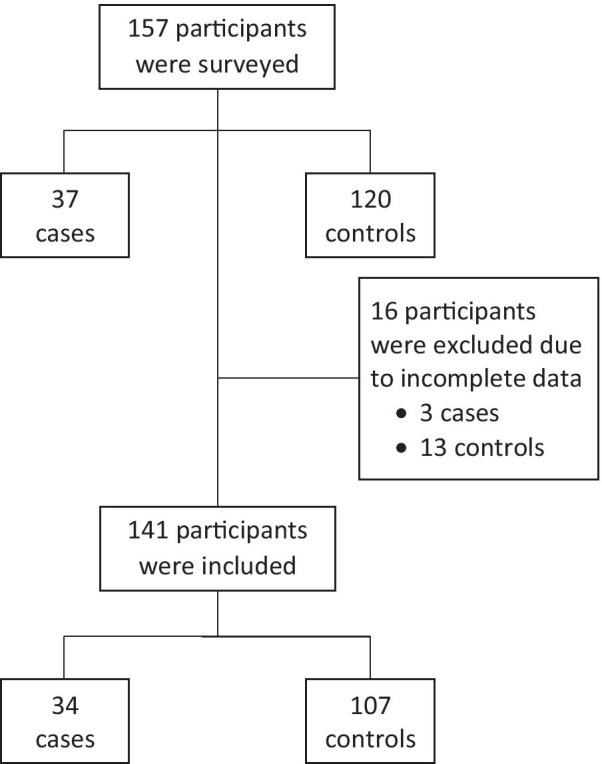
Flow diagram of the study population

Out of 157 participants, 16 (3 cases and 13 controls) were excluded due to missing data. Finally, 34 cases and 107 controls were included in the analysis (Fig. 2).

Seventy-six adolescents (53.9%) were 15 to 19 years old (late adolescence). The majority (85.1%) were native, studied high school (78%), and 72.3% were full-time students. Almost all (97.2%) were in the D–E socioeconomic status (< 300.00 USD as a monthly income), with 83% depending on their family. More than half of the participants (56.7%) ranked as a middle sibling. The mean age of menarche was 12.64 ± 1.06, while the mean age at first sexual intercourse was 14 ± 1.62. Of the adolescents who reported being sexually active, 33.3% used contraceptive methods. Furthermore, 77.3% had a family history of adolescent pregnancy. Nine cases desired to get pregnant (26.5%) and 53.2% reported sexual and reproductive health communication with their parents. More information related to family, parents’ and couple’s characteristics are presented in the Additional file 1.

Table 1 presents the bivariate analysis. Differences were observed in the age (p = 0.01), the educational level (p < 0.0001), parents’ educational level (p = 0.002, p = 0.006), and sexual and reproductive health communication with parents (p < 0.0001).

**Table 1:** Frequencies and percentages of adolescent pregnancy, according to sociodemographic, parents and family characteristics

Characteristics	Not adolescent pregnancy (n = 107)	Adolescent pregnancy (n = 34)	p-value
Age (years)			0.001^a^
10–14	58 (54.2)	7 (20.6)	
15–19	49 (45.8)	27 (79.4)	
Educational level			< 0.0001^b^
None	0 (0)	1 (2.9)	
Elementary school	15 (14)	15 (44.1)	
High school	92 (86)	18 (52.9)	
Socio-economic status			0.673^b^
A–B	1 (0.9)	0 (0)	
C	2 (1.9)	1 (2.9)	
D–E	104 (97.2)	33 (97.1)	
Adolescent's mother with a history of adolescent pregnancy			0.313^a^
No	64 (59.8)	17 (50)	
Yes	43 (40.2)	17 (50)	
Adolescent's father with a history of being an adolescent father			0.525^a^
No	90 (84.1)	27 (79.4)	
Yes	17 (15.9)	7 (20.6)	
Educational level of adolescent’s mother			0.002^b^
None	14 (13.1)	14 (41.2)	
Elementary school	66 (61.7)	18 (52.9)	
High school	23 (21.5)	2 (5.9)	
Technician/University	4 (3.7)	0 (0)	
Educational level of adolescent’s father			0.006^b^
None	6 (5.6)	8 (23.5)	
Elementary school	50 (46.7)	18 (52.9)	
High school	42 (39.3)	8 (23.5)	
Technician/University	9 (8.4)	0 (0)	
Sexual and reproductive health communication with parents			< 0.0001^a^
No	40 (37.4)	26 (76.5)	
Yes	67 (62.6)	8 (23.5)	
Family history of adolescent pregnancy			0.081^a^
No	28 (26.2)	4 (11.8)	
Yes	79 (73.8)	30 (88.2)	
Position within siblings			0.501^a^
Yungest	15 (14.1)	6 (17.6)	
Middle	59 (55.1)	21 (61.8)	
Oldest	33 (30.8)	7 (20.6)	
Number of siblings			0.38^a^
1–4	52 (48.6)	12 (35.3)	
5–6	27 (25.2)	10 (29.4)	
7–12	28 (26.2)	12 (35.3)	

^a^Chi-squared test

^b^Fisher's exact test

Table 2 displays the nested multivariable analysis. We reported a significant association of having 15 to 19 years old (OR = 6.88, 95% CI 2.38–19.86, p < 0.0001) and an elementary school level (OR = 5.59, 95% CI 1.95–16.06, p = 0.001) with the risk of adolescent pregnancy. A marginal statistical significance between having five to six siblings and adolescent pregnancy was also reported (OR = 2.70, 95% CI 0.85–8.61, p = 0.094). On the other hand, adolescents who had sexual and reproductive health communication with parents had a lower risk of adolescent pregnancy (OR = 0.17, 95% CI 0.06–0.47, p = 0.001).

**Table 2:** Factors associated with adolescent pregnancy among female indigenous adolescents from the Peruvian central jungle

Variables	Bivariate analysis			Multiple regression, Parsimonious Model^c^		
	OR^a^	95% CI^b^	p-value	OR	95% CI^b^	p-value
Age (years)						
10–14	Ref.			Ref.		
15–19	4.33	1.78–10.57	0.001	6.88	2.38–19.86	< 0.0001
Educational level						
None	14.99	0.59–382.74	0.101	2.17	0.07–63.53	0.652
Elementary school	5.00	2.11–11.85	< 0.0001	5.59	1.95–16.06	0.001
High school	Ref.			Ref.		
Socio-economic status						
A–B	Ref.			–	–	–
C	1.79	0.04–79.42	0.761	–	–	–
D–E	0.96	0.38–24.17	0.981	–	–	–
Adolescent's mother with a history of adolescent pregnancy						
No	Ref.			–	–	–
Yes	1.48	0.69–3.19	0.314	–	–	–
Adolescent's father with a history of being an adolescent father						
No	Ref.			–	–	–
Yes	1.41	0.54–3.67	0.481	–	–	–
Educational level of adolescent’s mother						
None	Ref.			–	–	–
Elementary school	0.96	0.04–23.48	0.979	–	–	–
High school	2.50	0.13–48.65	0.544	–	–	–
Technician/University	8.99	0.44–182.78	0.153	–	–	–
Educational level of adolescent’s father						
None	Ref.			–	–	–
Elementary school	3.80	0.20–71.71	0.373	–	–	–
High school	6.96	0.39–125.65	0.189	–	–	–
Technician/University	24.85	1.21–509.96	0.037	–	–	–
Sexual and reproductive health communication with parents						
No	Ref.			Ref.		
Yes	0.19	0.08–0.46	< 0.0001	0.17	0.06–0.47	0.001
Family history of adolescent pregnancy						
No	Ref.			–	–	–
Yes	2.43	0.83–7.14	0.107	–	–	–
Position within siblings						
Yungest	1.16	0.41–3.28	0.779	–	–	–
Middle	Ref.			–	–	–
Oldest	0.62	0.24–1.57	0.314	–	–	–
Number of siblings						
1–4	Ref.			Ref.		
5–6	1.60	0.63–4.11	0.325	2.70	0.85–8.61	0.094
7–12	1.84	0.74–4.56	0.187	2.51	0.81–7.78	0.109

^a^OR, odds ratio

^b^CI, Confidence Interval

^c^Nested Model estimated by manual forward selection

Table 3 shows the perception of adolescent pregnancy, with 80.3% opposing to sexual intercourse at an early age. Family (21.8%) and socioeconomic problems (20.3%) were the most frequent ones. Finally, only 7.3% pointed out employment issues concerning adolescent pregnancy.

**Table 3:** Perceptions towards adolescent pregnancy of female indigenous adolescents from the Peruvian Central Jungle

Question	n (%)
What is your attitude towards sexual intercourse at an early age? (n = 137)	
Disagree	110 (80.3)
Agree	27 (19.7)
Do you believe that adolescent pregnancy may carry trouble with…^a^ (n = 385)	
Health?	76 (19.7)
Emotions?	45 (11.7)
Education?	74 (19.2)
Socio-economic Status?	78 (20.3)
Employment issues?	28 (7.3)
Family?	84 (21.8)

^a^Multiple choice question

The post hoc power analysis for the four variables included in the parsimonious model obtained statistical powers greater than 90% for all cases, except for the number of siblings (< 80%).This could be explained because the initially calculated sample number could not be reached (Fig. 2). We decided to consider this variable in the discussion because it was selected through the manual forward selection process and it has been described in the literature as a determinant for adolescent pregnancy [27, 28].

## Discussion

In this study, we report that being 15 to 19 years old, having an elementary educational level, and sexual and reproductive health communication with parents are determinants of adolescent pregnancy in female indigenous adolescents from the Peruvian Central Jungle. In addition, a marginal statistical significance was found between having five to six siblings and adolescent pregnancy.

In the Peruvian context, these results are relevant due to adolescent pregnancy prevalence raised from 2016 (12.7%) to 2017 (13.4%), with higher values in jungle regions [29]. Furthermore, it was mainly distributed in rural areas, with 23% of female adolescents aged 15 to 19 years old in 2017 [30, 31]. Our results focus on a specific population from rural areas of the Peruvian Central jungle, which lack scientific data related to factors associated with adolescent pregnancy.

We report that late adolescence (15 to 19 years old) has a six-fold increase risk of adolescent pregnancy. Around the world, different studies have reported that the frequency of adolescent pregnancy was higher in the late adolescence group [21, 32–35]. Nevertheless, no significant difference between age groups was found in two African studies [9, 36], a discrepancy that may be linked with the sparse number of adolescents in the early adolescence group.Therefore, family planning interventions should be focused on female indigenous adolescents aged 15 to 19 years old to diminish the risk of adolescent pregnancy, without neglecting the early adolescence group.

In our study, an elementary educational level increased five times the risk of adolescent pregnancy. These results are similar to studies conducted in Asia [32, 37, 38] and Africa [36, 39], considering the higher educational component as a protective factor of repeated adolescent pregnancy [40]. In Latin America, a Colombian case–control study revealed that six out of ten pregnant adolescents coursed less than nine years of education, which was a lower time of education compared with controls. However, the educational level was not associated with adolescent pregnancy in the multivariate analysis [21]. A systematic review in low- and middle-income countries showed that elementary-level education was a common risk factor, especially in African countries [41]. The empowerment of women through education should be an effective strategy to avoid adolescent pregnancy since education seems to be a cornerstone of cultural development in different settings [42].

We found a marginal statistical significance between having five to six siblings and adolescent pregnancy. This variable needs further revision since we attribute this finding to our sample size that did not reach an adequate statistical power in the post hoc analysis. Some studiesreported similar results, although they have used a different approach to evaluate the number of siblings. For example, a study conducted in rural areas of South Africa, where the population on average had six members in their household, found that 44.4% of female adolescents had a history of pregnancy [43]. Similarly, a Canadian study reported that the history of adolescent pregnancy of an older daughter increased three times the risk of adolescent pregnancy in a younger sister [7]. Further research is needed to clarify the influence of this variable on adolescent pregnancy.

Unlikely other studies [34, 37–39], no significant association was found between the socioeconomic status and adolescent pregnancy, despite their higher vulnerability and geographic limitation. A South Asian systematic review showed that the incidence of adolescent pregnancy was significantly higher in households with low wealth index [44]. Similarly, an African study stated that this factor increased the risk of adolescent pregnancy up to two times than in those who came from more favored contexts [39]. Our results may be attributed to the similar socioeconomic condition of the studied population, with only four adolescents in A-B and C socioeconomic status.

Parents of seven out of ten cases did not talk about sexual and reproductive health with their daughters. This could be related to the fact that sexual and reproductive health communication with parents was a protective factor, diminishing in 83% the risk of adolescent pregnancy in our study. An African systematic review found similar results reporting that the lack of sexual and reproductive health communication increased two times the risk of adolescent pregnancy [45]. Cultural taboos may influence the restriction of sexual and reproductive health communication [35], a gap that schools should fill. However, the indigenous setting represents a challenge due to sexism and the lack of choice to become pregnant. Sexual health education should be reformulated, focusing on cultural barriers within indigenous communities [46]. This would contribute to indigenous women empowerment to overcome cultural barriers, guide sexual decision-making, and enhance the quality of life [47]. Further qualitative studies must address this topic in the Peruvian central jungle.

We inform that most female indigenous adolescents opposed to sexual intercourse at an early age. Also, they denoted that adolescent pregnancy may carry different consequences, especially within the family, socioeconomic, health, and educational environment. Nonetheless, they tend to have no choice related to pregnancy due to sexism and lack of empowerment, a subject that was out of the scope of this study. An Argentinean qualitative study that explored this topic reported that adolescents considered their age as an incorrect time for motherhood [46]. Likewise, Thai pregnant refugees and migrant adolescents considered 20 years old as the best age to have a child; however, they had been not completely able to choose when to conceive [48].

In our study, education was one of the most common issues reported as a problem of adolescent pregnancy. School abandonment was distinguished as one of the most common social consequences of adolescent pregnancy in different settings. For instance, a Colombian study found that six out of ten adolescent mothers considered school dropout as the main effect of adolescent pregnancy [49]. Furthermore, studies conducted in refugees and migrants setting [40], outpatient clinical setting [50], and African urban areas [51] reported that school abandonment was common among pregnant adolescents.

Finally, health consequences related to adolescent pregnancy are well reported in medical literature, with severe adverse events on newborn and maternal health [39, 45]. Female indigenous adolescents’ disagreement with the early onset of sexual intercourse and knowledge about the major consequences of adolescent pregnancy is an initial step to focus interventions. Nonetheless, cultural barriers should be faced carefully to make male indigenous people aware of these issues. Studies related to cultural factors and social consequences of adolescent pregnancy in indigenous communities are needed to direct interventions.

Some limitations must be highlighted. First, it was hard to apply a randomization technique due to the lack of official registries of adolescents within these communities and their constant absence because of farm work. Second, we had a small sample size, which is why we used a penalized likelihood approach in our regression model. Third, we were at risk of desirability bias; nevertheless, a team of female interviewers who explained the relevancy of this topic, the importance of their honest answers, and the anonymity of data aid to overcome this limitation. Likewise, amphoras helped to adolescents' participation and confidence. Fourth, the native language; however, we had the participation of a translator, whose role was only needed in the farthest community (Boca Sanibeni) with five participants. Finally, our results could not be extrapolated to indigenous communities of different regions due to the broad intercultural differences that exist between them. Nevertheless, this study could represent an important approximation to the reality of indigenous communities from jungle regions in Peru.

## Conclusions

In summary, we reported that 15 to 19 years old, the elementary educational level, and sexual and reproductive health communication with parents are determinants of adolescent pregnancy in indigenous communities of the Peruvian Central Jungle. Furthermore, we found a marginal statistical significance between having five to six siblings and adolescent pregnancy in this setting. Our results suggest that public health and educational efforts should be age-specific focused within indigenous communities of the Peruvian central jungle. Furthermore, sexual and reproductive health communication with parents must be promoted.

## Supplementary Information

**Additional file 1: **Socio-Demographic, family, sexual and couple characteristics of female indigenous adolescents from the Peruvian Central Jungle (n = 141).

## Data Availability

The datasets used and/or analyzed during the current study are available from the corresponding author on reasonable request.

## Abbreviations

SD: Standard deviation
IQR: Interquartile range
ORs: Odds Ratios
CI: Confidence Interval
CUMIS: Multidisciplinary University Research and Service Camp (Campamento Universitario Multidisciplinario de Investigación y Servicio).
SOCIEMC: Scientific Society of Medical Students from the Center of Peru (Sociedad Científica de Estudiantes de Medicina del Centro)
