# Supplementary material for: Determinants of adolescent pregnancy in indigenous communities from the Peruvian central jungle: a case–control study
Source: Reprod Health. 2021 Oct 12;18:203. doi: 10.1186/s12978-021-01247-z (PMC8507392; doi:10.1186/s12978-021-01247-z)
Supplement: Supplementary file 1 — Additional file 1: Socio-Demographic, family, sexual and couple characteristics of female indigenous adolescents from the Peruvian Central Jungle (n = 141). [file 12978_2021_1247_MOESM1_ESM.docx]

**Additional file 1** Socio-Demographic, family, sexual and couple characteristics of female indigenous adolescents from the Peruvian Central Jungle (n=141)

| **Characteristics** | **n (%)** |
| --- | --- |
| **Age (years)** |  |
| 10-14 | 65 (46.1) |
| 15-19 | 76 (53.9) |
| **Years*** | 15 (14-16) |
| **Place of birth** |  |
| Native | 120 (85.1) |
| Foreign | 21 (14.9) |
| **Educational level** |  |
| None | 1 (0.7) |
| Elementary school | 30 (21.3) |
| High school | 110 (78.0) |
| **Occupation** |  |
| None | 12 (8.5) |
| Study | 102 (72.3) |
| Work | 3 (2.1) |
| Study and work | 24 (17.0) |
| **Socioeconomic status** |  |
| A-B | 1 (0.7) |
| C | 3 (2.1) |
| D-E | 137 (97.2) |
| **Economic dependence** |  |
| By herself | 2 (1.4) |
| By her family | 117 (83.0) |
| By her couple | 14 (9.9) |
| By her family and couple | 8 (5.7) |
| **Position within siblings** |  |
| Youngest | 21 (14.9) |
| Middle | 80 (56.7) |
| Oldest | 40 (28.4) |
| **Number of siblings** |  |
| 1-4 | 64 (45.4) |
| 5-6 | 37 (26.2) |
| 7-12 | 40 (28.4) |
| **Number of siblings*** | 5 (4-7) |
| **Cohabiting** |  |
| Alone | 3 (2.1) |
| Her family | 116 (82.3) |
| Her couple | 14 (9.9) |
| Her family and couple | 8 (5.7) |
| **Age of menarche**† (n=132)** | 12.64 ± 1.06 |
| **Age at first sexual intercourse**‡ (n=75)** | 14 ± 1.62 |
| **Adolescence stage at first sexual intercourse‡ (n=75)** |  |
| Early adolescence | 48 (64.0) |
| Late adolescence | 27 (36.0) |
| **Number of sexual partners† (n=137)** |  |
| Cero | 62 (45.3) |
| One | 51 (37.2) |
| Two | 11 (8.0) |
| Three | 13 (9.5) |
| **Use of Contraceptive Method‡ (n=75)** |  |
| No | 25 (33.3) |
| Yes | 50 (66.7) |
| **Frequency of Contraceptive Method use†‡ (n=67)** |  |
| Never | 25 (37.3) |
| Sometimes | 22 (32.8) |
| Always | 20 (29.9) |
| **Type of Contraceptive Method‡ (n=75)** |  |
| None | 25 (33.3) |
| Barrier | 24 (32.0) |
| Hormonal | 16 (21.3) |
| Barrier and Hormonal | 10 (13.3) |
| **Desired pregnancy (n=34)** |  |
| No | 25 (73.5) |
| Yes | 9 (26.5) |
| **Abortion history** |  |
| No | 132 (93.6) |
| Yes | 9 (6.4) |
| **Number of pregnancies** |  |
| Zero | 107 (75.9) |
| One | 29 (20.6) |
| Two | 5 (3.6) |
| **Educational level of adolescent’s mother** |  |
| None | 28 (19.9) |
| Elementary school | 84 (59.6) |
| High school | 25 (17.7) |
| Technician/University | 4 (2.8) |
| **Educational level of adolescent’s father** |  |
| None | 14 (9.9) |
| Elementary school | 68 (48.2) |
| High school | 50 (35.5) |
| Technician/University | 9 (6.4) |
| **Family history of adolescent pregnancy** |  |
| No | 32 (22.7) |
| Yes | 109 (77.3) |
| **Adolescent's Mother with a history of adolescent pregnancy** |  |
| No | 81 (57.5) |
| Yes | 60 (42.6) |
| **Adolescent's Father with a history of being an adolescent father** |  |
| No | 117 (83.0) |
| Yes | 24 (17.0) |
| **Sexual and reproductive health communication with parents** |  |
| No | 66 (46.8) |
| Yes | 75 (53.2) |
| **Adolescent couple's age*†~ (n=33)** | 20 (18-22) |
| **Adolescent couple's educational level~ (n=34)** |  |
| None | 0 (0.0) |
| Elementary school | 7 (20.6) |
| High school | 24 (70.6) |
| Technician/University | 3 (8.8) |
| **Adolescent couple's place of birth~ (n=34)** |  |
| Native | 22 (64.7) |
| Foreign | 12 (35.3) |
| **Adolescent couple's occupation~ (n=34)** |  |
| None | 0 (0.0) |
| Study | 4 (11.8) |
| Work | 24 (70.6) |
| Study and work | 6 (17.7) |
| * Median (interquartile range)  ** Mean ± standard deviation  † Sample is lower because of missings  ‡ Data correspond to teenagers who had at least one sexual intercourse  ~ Data correspond to cases (n=34) | |
